# Prevention of Memory Impairment and Neurotrophic Factors Increased by Lithium in Wistar Rats Submitted to Pneumococcal Meningitis Model

**DOI:** 10.1155/2017/6490652

**Published:** 2017-10-22

**Authors:** Lutiana R. Simões, Roberta R. E. S. Abreu, Jaqueline S. Generoso, Jéssica A. Goularte, Allan Collodel, Vijayasree Vayalanellore Giridharan, Anitha Christy Sigamani Arumanayagam, Samira S. Valvassori, João Quevedo, Tatiana Barichello

**Affiliations:** ^1^Laboratory of Experimental Microbiology, Graduate Program in Health Sciences, Health Sciences Unit, University of Southern Santa Catarina, Criciúma, SC, Brazil; ^2^Translational Psychiatry Program, Department of Psychiatry and Behavioral Sciences, McGovern Medical School, The University of Texas Health Science Center at Houston (UTHealth), Houston, TX, USA; ^3^Department of Pathology, Houston Methodist Research Institute, Houston, TX 77030, USA; ^4^Laboratory of Neurosciences, Graduate Program in Health Sciences, Health Sciences Unit, University of Southern Santa Catarina, Criciúma, SC, Brazil; ^5^Center of Excellence on Mood Disorders, Department of Psychiatry and Behavioral Sciences, McGovern Medical School, The University of Texas Health Science Center at Houston (UTHealth), Houston, TX, USA; ^6^Neuroscience Graduate Program, The University of Texas MD Anderson Cancer Center UTHealth Graduate School of Biomedical Sciences, Houston, TX, USA

## Abstract

The aim of this study was to investigate the effects of lithium on brain-derived neurotrophic factor (BDNF), nerve growth factor (NGF), and glial cell line-derived neurotrophic factor (GDNF) expression in the hippocampus and on memory in experimental pneumococcal meningitis. The mood-stabilizer lithium is known as a neuroprotective agent with many effects on the brain. In this study, animals received either artificial cerebrospinal fluid or *Streptococcus pneumoniae* suspension at a concentration of 5 × 10^9^ CFU/mL. Eighteen hours after induction, all animals received ceftriaxone. The animals received saline or lithium (47.5 mg/kg) or tamoxifen (1 mg/kg) as adjuvant treatment, and they were separated into six groups: control/saline, control/lithium, control/tamoxifen, meningitis/saline, meningitis/lithium, and meningitis/tamoxifen. Ten days after meningitis induction, animals were subjected to open-field habituation and the step-down inhibitory avoidance tasks. Immediately after these tasks, the animals were killed and their hippocampus was removed to evaluate the expression of BDNF, NGF, and GDNF. In the meningitis group, treatment with lithium and tamoxifen resulted in improvement in memory. Meningitis group showed decreased expression of BDNF and GDNF in the hippocampus while lithium reestablished the neurotrophin expression. Lithium was able to prevent memory impairment and reestablishes hippocampal neurotrophin expression in experimental pneumococcal meningitis.

## 1. Introduction

Bacterial meningitis has resulted in significant rates of morbidity and mortality worldwide [1]. Despite the bacteria killed by antibiotics, cognitive impairment after pneumococcal meningitis is estimated to occur in about 30 to 52% of surviving patients. The most frequently reported sequelae are hearing loss, epilepsy, focal neurological deficits, and cognitive impairment [1–4].

A number of experimental studies have demonstrated the learning and memory impairment in rodent survivors after pneumococcal meningitis [5, 6]. The memory impairment showed a significant positive correlation with low brain-derived neurotrophic factor (BDNF) expression in the hippocampus of these rodents [7]. The BDNF, glial cell-derived neurotrophic factor (GDNF), and neurotrophin nerve growth factor (NGF) are substantial for the survival, maintenance, and regeneration of specific neuronal populations in the adult brain, and these neurotrophins play a crucial role in cognition, learning, and memory [8, 9].

The mood-stabilizer lithium is known as a neuroprotective agent with many effects on the brain. It is believed that lithium depletes inositol levels, inhibits glycogen synthase kinase- (GSK-) 3*β*, and other signaling molecules, neurotransmitters, and cellular pathways in the brain. The previous study reports that lithium also enhanced neurogenesis, promoted neuronal differentiation of progenitor cells [10–12], selectively activated BDNF promoter IV of primary neurons, and elevated BDNF levels in rat hippocampal, frontal, and temporal cortices [13, 14]. Lithium treatment prevented hippocampal apoptosis and improved spatial memory in experimental meningitis [15]. On the other hand, in experimental sepsis, lithium decreased proinflammatory cytokines and increased enzymatic antioxidant defense in the lung tissues of rats [16].

Besides lithium, the other drug that has protein kinase C (PKC) inhibitory feature is tamoxifen [17]. Tamoxifen is a lipophilic and estrogen receptor modulator and the only known brain permeable PKC inhibitor [18]. PKC belongs to a family of enzymes which phosphorylate neurotransmitter receptors, triggering intracellular signaling molecules, transcription factors, and cytoskeletal proteins. PKC knockout mice showed reduced inflammation in several inflammatory disorders such as asthma, multiple sclerosis, arthritis, and inflammatory bowel disease. Tamoxifen is also found to be neuroprotective in dopaminergic models of neurotoxicity [19] and in amyotrophic lateral sclerosis [20].

The combined antiproliferation, as well as anti-inflammation properties of GSK-3*β*, and PKC molecule inhibitors such as lithium and tamoxifen could open a new avenue in the adjuvant bacterial meningitis treatment. Hence, the aim of this study was to investigate the effects of lithium and tamoxifen on BDNF, GDNF, and NGF expression in the hippocampus and on behavior tasks in an experimental model of pneumococcal meningitis.

## 2. Materials and Methods

### 2.1. Infecting Organisms

The strain of serotype III *Streptococcus pneumoniae* was cultured in a 10 mL Todd Hewitt Broth, Himedia® and then diluted in fresh medium and grown to logarithmic phase. The culture was centrifuged during 10 min at 5000*g* and resuspended in sterile, pyrogen-free saline to the concentration of 5 × 10^9^ colony-forming units (CFU) [21].

### 2.2. Meningitis Animal Model

Male Wistar rats (8–10 weeks), weighing 200–250 g, were obtained from our breeding colony and used for the experiments. All procedures were approved by the Animal Care and Experimentation Committee of UNESC-Brazil sob protocol 045/2013 and 074/2015-1. Bacterial meningitis induction was performed under anesthesia consisting of an intraperitoneal injection of ketamine (6.6 mg/kg), xylazine (0.3 mg/kg), and acepromazine (0.16 mg/kg) [22]. The animals received intracisternal injection of 10 *μ*L of artificial cerebrospinal fluid (CSF) as a placebo or an equivalent volume of *S. pneumoniae* serotype III suspension, followed by 1 mL of fluid replacement subcutaneous. 18 hours after meningitis induction, the animals were anesthetized and 5 *μ*L of CSF was collected by puncturing the cisterna magna to confirm the meningitis infection. A quantitative culture of 5 *μ*L of CSF was incubated at 35°C with 5% CO_2_ on 5% sheep blood agar [21]. All infected rodents presented positive culture to *S. pneumoniae*.

### 2.3. Treatment

**Figure 1:**
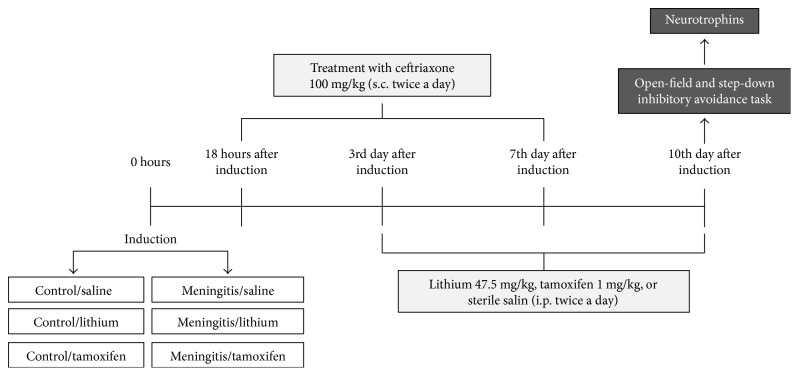
Timeline outline of the induction of meningitis, the adjuvant treatments, and the tests performed.

Eighteen hours after meningitis induction or artificial CSF inoculation, all animals received ceftriaxone (100 mg/kg s.c. for 7 days) [22]. Seventy-two rats were divided in six groups: control/saline, control/lithium, control/tamoxifen, meningitis/saline, meningitis/lithium, and meningitis/tamoxifen. Lithium (47.5 mg/kg i.p. twice a day), tamoxifen (1 mg/kg i.p. twice a day), or sterile saline was administered from day 3 to day 10 after meningitis or artificial CSF inoculation [23] (Figure 1).

### 2.4. Behavioral Tasks

Ten days after meningitis induction, the animals were free from infection. All cultures were negative (data not shown). Then, the animals were randomized and subjected to the following behavioral tests: habituation to the open-field and step-down inhibitory avoidance tasks.

#### 2.4.1. Open-Field Task

The apparatus was a 40 × 60 cm open field surrounded by 50 cm high dark grey walls and a front glass wall. Black lines divided the floor of the open field into nine rectangles. Each animal was gently placed in the center of the open field and was left to explore the arena for 5 min (training session). The numbers of crossings (the number of times that the animal crossed the black lines, an assessment of locomotor activity) and rearing movements (the exploratory behavior was observed in rats subjected to a new environment) were measured. Immediately after this procedure, the animals were taken back to their home cage. Twenty-four hours later, the animals were subjected to a test session then the number of times the animal crossed the black lines or reared was counted during a 5 min period. The reduction in the number of crossings and rearings between the two sessions was taken as a measure of the retention of memory. The behavioral test was performed by the same person who was blind to the group treatment [24].

#### 2.4.2. Step-Down Inhibitory Avoidance Task

The apparatus consists in a 50 × 25 × 25 cm acrylic box (Albarsch, Porto Alegre, Brazil), whose floor consisted of parallel stainless steel bars (1 mm diameter) and is spaced 1 cm apart. A 7 cm wide, 2.5 cm high platform was placed on the floor of the box, against the left wall. In the training trial, animals were placed on the platform, and their latency to step down on the grid with all four paws was measured with an automatic device. Immediately after stepping down on the grid, the animals received a 0.4 mA for 2.0 s foot shock and were returned to their home cage. A retention test trial was performed 1.5 h (short-term memory) and 24 h after training (long-term memory). The retention test trial was procedurally identical to the training trial, except that no foot shock was administered. The retention test step-down latency (maximum, 180 s) was used as a measure of inhibitory avoidance retention [25–27].

### 2.5. Assessment of BDNF, NGF, and GDNF Expression

After behavioral tasks, the animals were euthanized by decapitation and their hippocampus was rapidly dissected and immediately stored at −80°C. BDNF, NGF, and GDNF expressions were evaluated using sandwich enzyme-linked immunosorbent assay (ELISA) according to the manufacturer's instructions (NGF and BDNF from Chemicon, USA and GDNF from Biosensis, USA). Microtiter plates (96-well flat-bottom) were coated for 24 h with the samples diluted 1 : 2 in sample diluent, and standard curves ranging from 7.8 to 500 pg of BDNF or NGF were established. Plates were then washed four times with sample diluents. Monoclonal anti-BDNF rat antibody, monoclonal anti-NGF rat antibody, or polyclonal rat GDNF antibody diluted 1 : 1.000 in sample diluent was incubated for 3 h at room temperature. After washing, the second incubation with anti-rat antibody peroxidase conjugated diluted 1 : 1.000 for 1 h at room temperature was carried out. After the addition of streptavidin-enzyme, substrate, and stop solution, the amount of BDNF or NGF was determined with an absorbance of 450 nm. The standard curve demonstrated a direct relationship between optical density (OD) and BDNF, NGF, and GDNF concentrations. The protein was measured by Lowry's method (1951) [28] using bovine serum albumin as a standard, as previously described by Frey et al. (2006) [14].

## 3. Statistics

The data were analyzed for normality using the Shapiro-Wilk test and for homogeneity using the Levene test. If the data were normal and homogeneity of variance was confirmed, parametric tests were used; if the data did not meet this condition, nonparametric tests were used. For the neurotrophin analyses, the data were reported as the mean ± SEM and was analyzed by two-way ANOVA, followed by the Tukey post hoc test. Data from the habituation to open-field task were reported as the mean ± SD, and the groups were compared using paired Student's *t*-test and analysis of variance followed by Tukey's post hoc test. Comparisons among groups for the step-down inhibitory avoidance task were performed using a Mann–Whitney *U* test. The intragroup comparisons were performed using Wilcoxon's tests. In all comparisons, *P* < 0.05 indicated statistical significance. All analyses were performed using the Statistical Package for the Social Science (SPSS) software version 20.0.

## 4. Results

**Figure 2:**
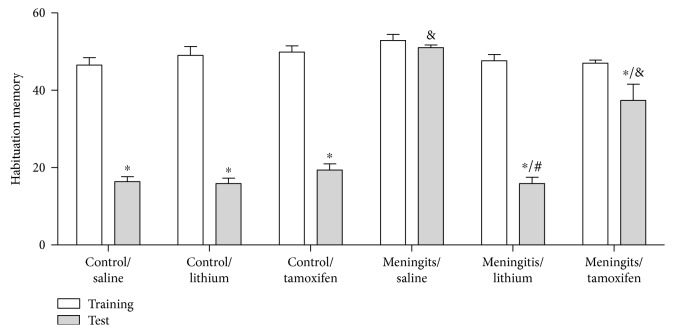
Effects of lithium and tamoxifen on habituation to open-field task in adult Wistar rats 10 days after pneumococcal meningitis induction. The numbers of crossings and rearing movements are reported as the mean ± SD and were analyzed by ANOVA and post hoc Tukey's tests (*n* = 12). ^∗^*P* < 0.05 statistically different from training session; ^&^*P* < 0.05 different from control/saline group; and ^#^*P* < 0.05 different from meningitis/saline group.

**Figure 3:**
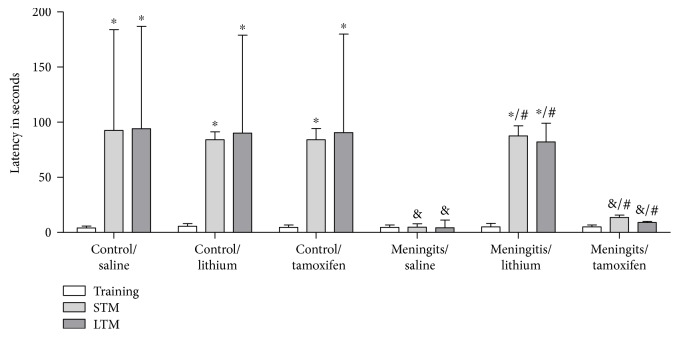
Effects of lithium and tamoxifen on step-down inhibitory avoidance task in adult Wistar rats 10 days after pneumococcal meningitis induction. Data are reported as median and interquartile ranges, and comparisons among groups were performed using Mann–Whitney *U* tests (*n* = 12). The within-individual groups were analyzed by Wilcoxon's tests. ^∗^*P* < 0.05 indicates differences between training and test sessions as determined by Wilcoxon, ^&^*P* < 0.05 indicates the statistical significance of the control/saline group as described by Mann–Whitney, and ^#^*P* < 0.05 indicates statistical significance of the meningitis/saline group as determined by Mann–Whitney.

We investigated the effects of lithium and tamoxifen on behavioral tasks of adult Wistar rats subjected to pneumococcal meningitis (Figures 2 and 3). We observed that meningitis group subjected to the open-field habituation task exhibited no difference between training and test sessions, demonstrating memory impairment. Moreover, 7 days of treatment with lithium and tamoxifen significantly improved memory in the open-field habituation task (*P* < 0.05; Figure 2).

Next, we investigated the effect of lithium and tamoxifen on step-down inhibitory avoidance task of rats subjected to pneumococcal meningitis. In the meningitis/saline and meningitis/tamoxifen groups, there were no differences between the training and test sessions, demonstrating impairment of short- and long-term aversive memories in these groups. Lithium treatment resulted in memory improvement, reflected in the statistically significant better performance observed in step-down inhibitory avoidance task after 7 days of lithium treatment (*P* < 0.05; Figure 3).

**Figure 4:**
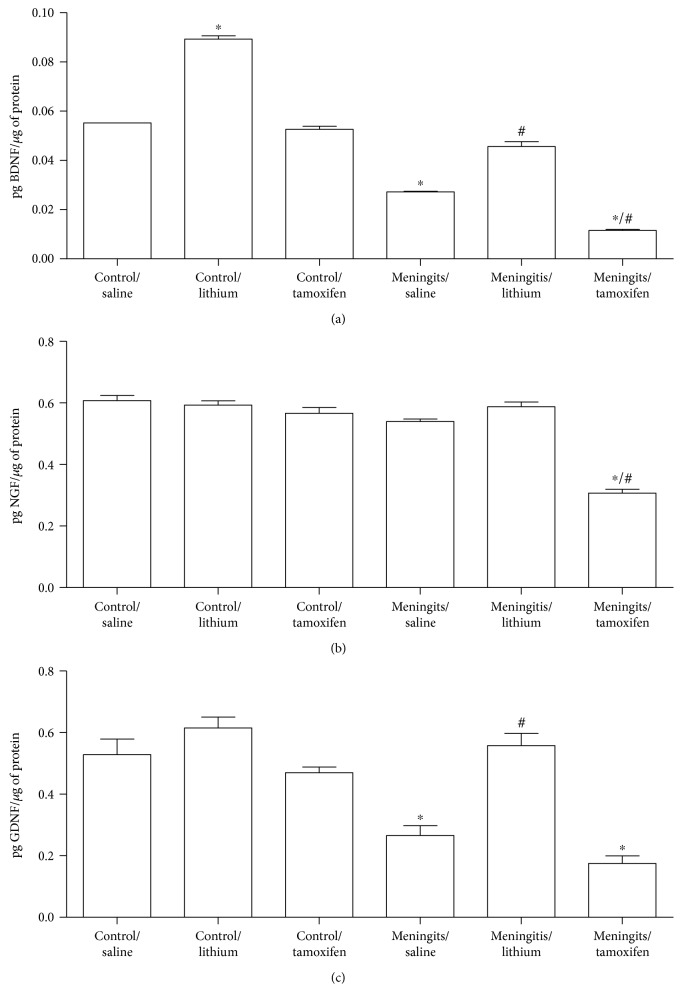
Effects of lithium and tamoxifen on BDNF, NGF, and GDNF expression in the hippocampus 10 days after pneumococcal meningitis induction. BDNF (a), NGF (b), and GDNF (c) levels were assessed by ELISA, and the results are presented in pg per *μ*g of protein, with *n* = 5 per group. All data are reported as the means ± SEM. These results were analyzed by two-way ANOVA, followed by the Tukey post hoc test. ^∗^*P* < 0.05 indicates the statistical significance of the control/saline group and ^#^*P* < 0.05 indicates the statistical significance of the meningitis/saline group.

After, we evaluated the effects of lithium and tamoxifen treatments on BDNF, NGF, and GDNF in the rat hippocampus. In the hippocampus, there were effects for meningitis (*F* = 167.43; *P* < 0.001) for treatments (*F* = 53.30; *P* < 0.001), as well as interaction (*F* = 2.80; *P* = 0.080) in the BDNF. BDNF expression decreased in the hippocampus of the meningitis/saline and meningitis/tamoxifen groups when compared with the control/saline group (*P* < 0.05). However, lithium as adjuvant treatment reestablished BDNF expression in the meningitis group (Figure 4(a)). In NGF expression in the hippocampus, there were effects for meningitis (*F* = 17.07; *P* < 0.001) for treatments (*F* = 13.15; *P* < 0.001), as well as interaction (*F* = 8.11; *P* = 0.002). NGF expression decreased in the meningitis/tamoxifen group when compared to the control/saline and meningitis/saline (*P* < 0.05; Figure 4(b)) groups. In the hippocampus, there were effects for meningitis (*F* = 50.64; *P* < 0.001) for treatments (*F* = 29.84; *P* < 0.001), as well as interaction (*F* = 6.65; *P* = 0.005) in the GDNF expression. GDNF expression decreased in the hippocampus of the meningitis/saline and meningitis/tamoxifen groups when compared with the control group (*P* < 0.05). Lithium reestablished GDNF levels in the meningitis group (Figure 4(c)).

## 5. Discussion

Pneumococcal meningitis has caused the highest fatality rate of 30%, and nearly 50% of survival patients showed cognitive impairment. The cognitive impairment as the result of the neuronal loss during infection occurs in the dentate gyrus of the hippocampus. Meningitis infection usually targeted this region associated with cognitive functions such as learning, hearing, seizures, motor handicaps, and other sequelae [29].

Any changes or alterations of neurotrophic factor level such as BDNF, NGF, and GDNF are related to cognitive deficit or impairment. Neurotrophic factors are nothing but secreted proteins that played an important role in the synaptic and neuronal growth, pruning, myelination, differentiation, and survival of neurons [30, 31]. Deficits of GDNF, NGF, and BDNF are related to disease pathology and symptoms, and their irreversible impairments are considered as potential therapeutics [32]. Several drugs act as neuroprotective agents by downregulating host inflammation, promoting anti-inflammatory cytokines, or inhibiting proinflammatory cytokines; leukocyte recruitment decreases these sequelae in experimental models as well as in patients [29]. Among those drugs, we studied the effect of tamoxifen as well as lithium on BDNF, GDNF, and NGF expression in the hippocampus and on behavior tasks in an experimental model of pneumococcal meningitis in rats.

In spite of the fact that tamoxifen acts as a potent estrogen agonist, several experimental studies showed that tamoxifen plays neuroprotective roles in spinal cord injury, intracerebral hemorrhage, brain ischemia, and hypoxic-ischemic brain injury. Several *in vitro* and i*n vivo* studies confirmed that tamoxifen can also induce anti-inflammatory response in acute models of mouse and rat primary hippocampal microglial cells by modulating proinflammatory signaling cascades [33]. However, in our experiment, we noticed tamoxifen decreased hippocampal BDNF, NGF, and GDNF expressions.

Lithium has been used in the treatment of manic depression for over 100 years [34]. This old drug has been studied as a neuroprotective agent in a variety of neurological, degenerative disorders and brain infections [15, 34, 35]. Indeed, these neuroproperties of lithium are associated with a stimulation of cell survival factors suggesting that lithium treatment enhances BDNF expression and secretion, leading to the activation of TrKB receptor [34, 36]. Earlier studies have documented that lithium is known to enhance neurogenesis and promote neuronal differentiation of progenitor cells [10, 12, 37]. In addition, *in vitro* lithium application and downregulation of GSK-3*β* resulted in increased proliferation of adult hippocampal progenitors [38]. The hippocampus is imperative for episodic and spatial memory and is implicated in emotional behavior and spatial memory [39, 40]. Knockout mice for DIX domain containing-1, an intracellular Wnt/*β*-catenin signal pathway protein, exhibit symptoms of anxiety, depression, and social behavior. Furthermore, these animals' brains presented pyramidal neurons with dendritic spines and reduced glutamatergic synapses [41, 42]. In a study by Martin et al. [42], the injection of either lithium chloride or a GSK-3 inhibitor altered behavioral phenotypes in Dixdc1 knockout mice in forced swim and social interactions in paired tasks. Moreover, it was observed that both lithium and GSK-3 inhibitor reestablished glutamatergic and dendritic spine synapses density in pyramidal neurons L5/6 in the brains of animals in Dixdc1 knockout mice. In our experiment, we observed that lithium prevented memory impairment and increased hippocampal BDNF, NGF, and GDNF neurotrophin expression in experimental pneumococcal meningitis.

Both lithium and tamoxifen improved memory in the experimental model of pneumococcal meningitis. But lithium reestablished hippocampal BDNF, NGF, and GDNF expression; however, tamoxifen adjuvant treatment did not show the same effect.

Thus, the present study demonstrated that adjuvant treatment with lithium prevented short-term and long-term aversive memory impairments, as well as open-field habituation memory, while also reestablishing BDNF, NGF, and GDNF expression in the hippocampus of an experimental rodent model of pneumococcal meningitis. Similar results were found in other studies where adjuvant treatment with sodium butyrate reestablished BDNF and GDNF in the hippocampus and prevented behavioral damage [43].

## 6. Conclusion

Currently, improvements in treatment depend on a better understanding of the pathogenesis and pathophysiology of this disease. New experimental strategies are essential to decrease long-term cognitive impairment in survivors after meningitis. The present research showed that lithium prevented cognitive impairment through reestablishment of BDNF, NGF, and GDNF expression.
